# Phylogeography and paleodistribution models of a widespread birch (*Betula platyphylla* Suk.) across East Asia: Multiple refugia, multidirectional expansion, and heterogeneous genetic pattern

**DOI:** 10.1002/ece3.5365

**Published:** 2019-06-14

**Authors:** Tian‐Yi Chen, An‐Ru Lou

**Affiliations:** ^1^ Ministry of Education Key Laboratory for Biodiversity Science and Ecological Engineering, College of Life Sciences Beijing Normal University Beijing China

**Keywords:** East Asia, ecological niche modeling, gene flow, genetic diversity, migration, quaternary oscillations

## Abstract

Widespread tree species cover large geographical areas and play important roles in various vegetation types. Understanding how these species responded to historical climatic changes is important for understanding community assembly mechanisms with evolutionary and conservation implications. However, the location of refugial areas and postglacial history of widespread trees in East Asia remain poorly known. We combined microsatellite data (63 populations, 1756 individuals) and ecological niche modeling to examine the range‐wide population diversity, genetic structure, and historical demography of a pioneer tree species, Asian white birch (*Betula platyphylla* Suk.) across East Asia. We found a north‐to‐south trend of declining genetic diversity and five clusters, corresponding to geographical regions. Different clusters were inferred to have diverged through Pleistocene climatic oscillations and have different expansion routes, leading to genetic admixture in some populations. Ecological niche models indicated that the distribution of *B. platyphylla* during the last glacial maximum still had a large latitude span with slight shifts toward southeast, and northern populations had more variable distribution ranges than those in the south during later climatic oscillations. Our results reflect the relatively stable distribution through the last glacial–interglacial cycles and recent multidirectional expansion of *B. platyphylla*, providing new hypotheses for the response pattern of widespread tree species to climate change. The gradual genetic pattern from northeast to southwest and alternative distribution dynamics possibly resulted from environmental differences caused by latitude and topographic heterogeneity.

## 1 INTRODUCTION

Climatic changes may cause habitat fragmentation, migration, adaption in plant populations or even promote extinction or speciation (Aitken, Yeaman, Holliday, Wang, & Curtis‐McLane, 2008; Yesson, Toomey, & Culham, 2009). The study of historical dynamics and genetic structures of different plants can help us understand the response patterns and survival conditions for specific species (Pearson et al., 2006). Previous studies have shown that climatic oscillations in the Quaternary period (<2 million years before present) led to periodic glacial/interglacial cycles, which profoundly affect genealogical differentiation, genetic diversity, and distribution in plant species (Hewitt, 2000; Qiu, Fu, & Comes, 2011). Combining fossil evidence, molecular data, and species paleodistribution modeling, researchers have investigated the effects of historical geological events and climate change on many plants, located refugial areas, reconstructed recolonization routes, and established models such as the southern refugia model, cryptic refugia, and refugia within refugia (Gavin et al., 2014; Gomez & Lunt, 2007; Gugger, Ikegami, & Sork, 2013; Ortego, Riordan, Gugger, & Sork, 2012; Souto, Kitzberger, Arbetman, & Premoli, 2015; Stewart & Lister, 2001; Stewart, Lister, Barnes, & Dalen, 2010). However, there are few studies of the effect of past shifts in climate on the species with large distributions, and the population history and postglacial migration pattern of widespread species remain unclear. Widespread tree species cover a large geographical distribution area, occupy a variety of landscapes, and play an important role in diverse plant communities. Clarifying the response pattern of these species to historical climatic changes is critical for understanding species and community dynamics on a large geographical scale, as well as providing suggestions for future conservation and management (Oberle & Schaal, 2011).

East Asia, which currently harbors the greatest temperate floral diversity (Liu, 1988; Qian & Ricklefs, 2001), is attracting more attention about the response pattern of plants to historical climatic change (Harrison, Yu, Takahara, & Prentice, 2001; Ni, Yu, Harrison, & Prentice, 2010; Qian & Ricklefs, 2000, 2001; Qiu et al., 2011). Paleontological reconstruction of East Asia based on fossil data suggests that, although East Asia was not covered by extensive glacial sheets (Liu, 1988; Shi, Ren, Wang, & Derbyshire, 1986), the evolution and distribution of plants were impacted by the chilliness and drought during the last glacial maximum (LGM, 21 000 years before present, kyr BP) (Cao, Herzschuh, Ni, Zhao, & Bohmer, 2015; Harrison et al., 2001; Yu et al., 2000). A widely held view about East Asia is that temperate deciduous forests were restricted to the southern region (25°–30°N) during the LGM based on pollen data (Harrison et al., 2001; Yu et al., 2000). However, researchers have questioned this view recently (Qian & Ricklefs, 2001; Qiu et al., 2011). Temperate deciduous trees might have survived the glacial periods in multiple northern refugia (around and above 40°N) (Bai, Liao, & Zhang, 2010; Wang, Xu, Zhang, & Bai, 2016; Wang et al., 2017; Zeng, Wang, Liao, Wang, & Zhang, 2015). In addition, East Asia has a complex relief. The elevation decreases from west to east; plains, mountains, basins, and highlands scatter this large area; southwest China also has the Qinghai–Tibetan Plateau (QTP) and the Hengduan Mountains, which contain a variety of high mountains and deep valleys. Regions with complex topography were thought to be rich in diverse microhabitats and environments, which can buffer climate fluctuations, providing great opportunities for plants to stay in situ or migrate out of the region (Birks, 2015; Qiu et al., 2011; Taberlet & Cheddadi, 2002). Hence, the phylogeographical patterns of species in southern and southeastern China are characterized by a strong effect of geography of those mountains and valleys, which provides multiple independent refugia for different species (Qiu et al., 2011). This conclusion is supported by the results of phylogeographical studies conducted on *Castanopsis eyrei* (Shi, Michalski, Welk, Chen, & Durka, 2014), *Primula secundiflora* (Wang, Gong, Hu, & Hao, 2008), *Fagus engleriana* (Lei et al., 2012), and *Platycarya strobilacea* (Chen et al., 2012). However, these studies focused on relatively narrow distribution ranges and the detailed historical processes involved in species located both in the north and in the south remain poorly understood (Guo et al., 2014; Liu et al., 2014).

Asian white birch (*Betula platyphylla* Suk.) is one of the most widely distributed temperate deciduous tree species in East Asia, mainly distributed in China, Russia, Korea, and Japan. It occurs primarily throughout the mountainous area extending from northeast to southwest, with an elevation span from 20 m to 4100 m (Chen, Manchester, & Sun, 1999; Zhang, Yang, Yu, & Shi, 2002). These mountains provide complex topography and various habitats for the species, making it one of the components of high‐cold meadow and steppe, temperate mixed conifer–deciduous broadleaved forest, temperate steppe, and subtropical evergreen broadleaved forest. *Betula platyphylla* is wind‐pollinated, and its pollen can be transported by air thousands of kilometers away (Sofiev, Siljamo, Ranta, & Rantio‐Lehtimaki, 2006). White birch is characterized by rapid growth and strong adaptability to environments, so it can grow as a pioneer tree species after disturbances, such as harvesting and forest fires, providing potential microhabitats for herbs and contributing to the stability and sustainability of forest ecosystems (Shi, Li, Koike, & Nie, 2001; Zyryanova, Terazawa, Koike, & Zyryanov, 2010). In addition to being an important successional tree in the restoration of vegetation, *B. platyphylla* is also an important economic tree species for timber production and tree sap utilization (Shi et al., 2001).

Studies on phylogeography of European *Betula* species indicated that, unlike most European species that survived the LGM in southern Europe, cold‐tolerant species, such as birches, inhabited glacial refugia at high latitudes (Jadwiszczak, 2012; Palme, Su, Rautenberg, Manni, & Lascoux, 2003; Willis & van Andel, 2004). More recently, a study of the genetic structure of *Betula* across Eurasia suggested that, in the *B. pendula*‐*B. platyphylla* species pair, genetic diversity was the highest in Siberia where hybrid populations were located, and *B. platyphylla* could have undergone rapid recolonization from Beringia or northeastern to central Siberia (Tsuda, Semerikov, Sebastiani, Vendramin, & Lascoux, 2017). However, this study only covered the populations of *B. platyphylla* at higher latitudes and did not include the populations in the East Asian continent. Evidence from a fossil pollen data set showed that birch trees in eastern continental Asia had four major distribution centers located on Changbai Mountain and adjacent areas, the mountainous areas of north‐central China, the southeastern Tibetan Plateau and the northwestern study region, and that postglacial expansion from these distribution centers to the surrounding areas showed no distinct expansion direction (Cao et al., 2015). In general, previous studies suggested the possible existence of northern refuge and multiple refugia for *B. platyphylla*; nevertheless, no phylogeographical study has performed a detailed reconstruction of glacial and postglacial history of this widespread species in East Asia. Further insights into the response patterns of widespread species to climate change can enhance our understanding of historical population dynamics and diversity maintenance.

**Figure 1:**
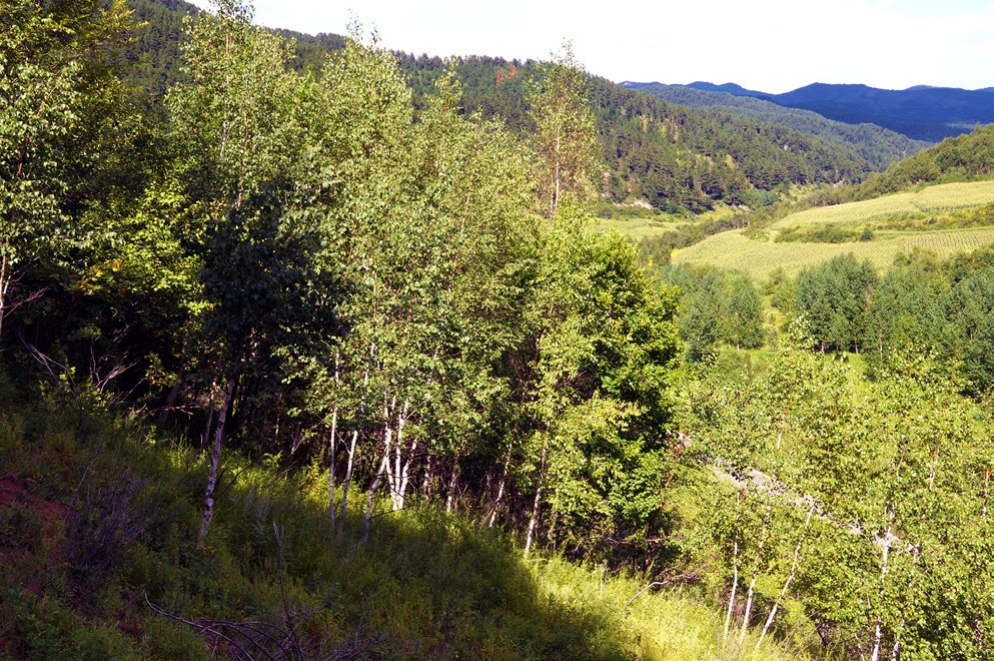
*Betula platyphylla* Suk

In this study, we used a specieswide analysis of nuclear microsatellite (nSSR) markers combined with ecological niche modeling approaches to investigate the phylogeographical structure and demographic history of *B. platyphylla* Suk. in East Asia. Our main goals were to (a) detect wide‐range genetic structure and patterns of genetic diversity of *B. platyphylla*, (b) infer the existence and locations of refugia for *B. platyphylla* during Quaternary climatic changes, and (c) reconstruct the glacial–postglacial history and migration pattern of this species. We tested the following hypotheses: If the refuge system of *B. platyphylla* corresponded to the southern refuge model, genetic footprints, such as a south‐to‐north decline in population genetic diversity corresponding with the direction of northward recolonization and extensive bottlenecks, should be expected; if the northern refuge model was supported, *B. platyphylla* presumably survived in both northern and southern refugia across East Asia and expanded from these refugia resulting in lineage admixture and secondary contact zones (Figure 1).

## 2 MATERIALS AND METHODS

### 2.1 Plant sampling

**Figure 2:**
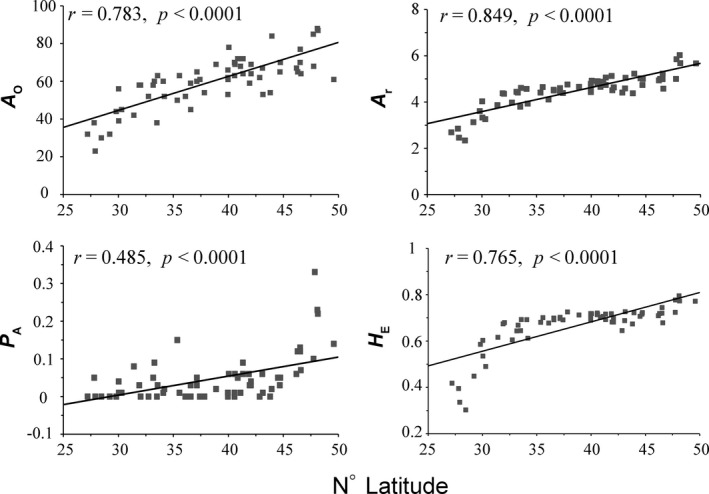
Correlations between latitude and intrapopulation nuclear diversity statistics of *Betula platyphylla*. *A*_O_, observed allele number; *A*_r_, allele richness; *P*_A_, private allelic richness; *H*_E_, expected heterozygosity

A total of 1756 wild white birch trees were sampled from 63 different geographical sites, including 16 from northeast China, 18 from north China, 11 from northwest China, 16 from southwest China, one from Japan, and one from Russian Far East (Table 1 and Figure 2). For each population, samples were collected from 8 to 41 individuals (mean = 28). Leaf tissues (one to three leaves per tree) were collected from trees at least 20 m apart from each other, dried with silica gel, and stored at room temperature before DNA extraction.

**Table 1:** Details of sample locations, sample sizes, and descriptive statistics of genetic variability for 63 populations of *Betula platyphylla*

Code	Sampling location	Longitude (°E)	Latitude (°*N*)	*n*	*A*_O_	*A*_r_	*P*_A_	*H*_E_	*F*_IS_	Bottleneck (TPM)
RA	Raohe1, Heilongjiang	133.66	46.53	28	77	5.23	0.12	0.744	0.076	0.988
RB	Raohe2, Heilongjiang	133.82	46.52	27	70	4.99	0.13	0.721	0.096	0.615
HN	Huanan, Heilongjiang	131.14	46.30	19	65	5.00	0.12	0.719	0.066	0.348
JM	Jiamusi, Heilongjiang	130.46	46.59	33	64	4.58	0.07	0.679	−0.013	0.993
MD	Mudanjiang, Heilongjiang	129.53	44.68	33	65	4.74	0.03	0.716	0.018	0.161
HL	Hailin, Heilongjiang	129.22	44.72	32	70	5.00	0.05	0.723	0.049	0.461
YC	Yichun, Heilongjiang	128.90	47.75	27	68	5.00	0.10	0.724	0.067	0.784
BY	Bayan, Heilongjiang	127.56	46.19	31	67	4.94	0.06	0.710	0.027	0.688
AT	Antu, Jilin	128.85	43.09	32	67	4.59	0.06	0.689	0.015	0.577
CB	Mt. Changbai, Jilin	127.67	42.06	31	69	4.63	0.04	0.682	0.006	0.947
JH	Jiaohe, Jilin	127.39	43.80	30	54	4.38	0.00	0.674	0.038	0.042*
HD	Huadian, Jilin	127.13	42.85	32	62	4.41	0.00	0.645	0.012	0.862
LJ	Linjiang, Jilin	127.03	41.93	30	59	4.51	0.03	0.682	0.057	0.615
QY	Qingyuan, Liaoning	125.29	42.05	30	64	4.64	0.03	0.698	0.088	0.754
HQ	Huanren1, Liaoning	125.12	41.10	30	68	4.88	0.01	0.706	0.07	0.884
HR	Huanren2, Liaoning	124.95	41.33	27	64	4.74	0.06	0.693	0.053	0.862
LH	Liaoheyuan, Hebei	118.45	41.33	30	72	5.06	0.09	0.717	0.037	0.577
WC	Mulanweichang, Hebei	117.67	41.86	31	77	5.13	0.06	0.718	0.034	0.784
WL	Mt. Wuling1, Hebei	117.49	40.56	29	70	4.90	0.00	0.714	0.034	0.884
WM	Mt. Wuling2, Hebei	117.48	40.56	22	63	4.81	0.01	0.721	−0.034	0.652
WN	Mt Wuling3, Hebei	117.46	40.60	28	69	4.89	0.06	0.715	0.057	0.754
XW	Mt. Xiaowutai, Hebei	115.31	39.96	21	61	4.79	0.00	0.719	0.001	0.784
CF	Chifeng, Neimenggu	117.51	43.94	40	84	5.23	0.02	0.722	0.062	0.947
BE	Hulunbeier, Neimenggu	117.48	49.58	10	61	5.67	0.14	0.772	0.002	0.216
XL	Xilinhaote, Neimenggu	116.87	44.50	30	76	5.03	0.05	0.707	0.021	0.988
DQ	Mt. Daqing, Neimenggu	111.27	40.85	39	72	4.66	0.05	0.703	0.063	0.615
DSS	Mt. Dongling, Beijing	115.49	40.04	34	78	4.92	0.06	0.718	0.009	0.947
GS	Xiaolongmengoushicao, Beijing	115.44	39.96	24	66	4.72	0.01	0.708	−0.013	0.539
DS	Xiaolongmennangou, Beijing	115.43	39.96	25	53	4.43	0.01	0.700	−0.16	0.007*
NGC	Xiaolongmennangoucha, Beijing	115.43	39.96	26	62	4.49	0.01	0.690	0.063	0.615
WT	Mt. Wutai, Shanxi	113.64	38.88	41	69	4.66	0.02	0.712	0.058	0.652
HS	Heshun, Shanxi	113.26	37.41	36	61	4.39	0.00	0.691	0.06	0.920
PQ	Pangquangou, Shanxi	111.47	37.82	31	54	4.76	0.00	0.726	0.104	0.216
WU	Mt. Wulu, Shanxi	111.20	36.58	8	45	4.50	0.00	0.692	−0.03	‐
BM	Mt. Baotianman, Henan	111.94	33.50	8	38	3.80	0.00	0.645	0.089	‐
FX	Fuxian, Shaanxi	109.68	36.09	33	52	4.11	0.00	0.678	−0.015	0.246
YN	Fengxian, Shaanxi	106.84	34.18	26	52	3.93	0.02	0.612	−0.143	0.754
KT	Mt. Kongtong, Gansu	106.43	35.56	33	63	4.65	0.01	0.701	0.071	0.688
HX	Huixian, Gansu	105.73	34.08	29	60	4.57	0.01	0.692	0.028	0.884
DB	Datong2, Qinghai	101.57	37.13	31	60	4.48	0.03	0.697	0.01	0.577
DA	Datong1, Qinghai	101.57	37.14	33	65	4.60	0.05	0.702	0.025	0.920
YS	Yaoshuihe, Qinghai	101.21	36.56	33	59	4.53	0.03	0.699	0.077	0.754
HB	Habahe, Xinjiang	86.22	47.88	30	85	5.81	0.33	0.776	0.102	0.423
XB	Qiba'er, Xinjiang	86.40	48.14	34	87	5.68	0.22	0.773	0.082	0.839
XA	Kulebai, Xinjiang	86.35	48.09	30	88	6.03	0.23	0.795	0.137	0.500
WB	Wangbachu, Sichuan	104.32	32.74	35	52	3.98	0.00	0.605	0.061	0.722
JZ	Jiuzhai, Sichuan	103.91	33.16	26	58	4.44	0.05	0.644	0.014	0.188
SX	Shenxianchi, Sichuan	103.73	33.28	30	60	4.41	0.09	0.669	0.058	0.995
DL	Daluxiang, Sichuan	103.67	33.57	31	63	4.61	0.03	0.693	0.059	0.500
LX	Lixian, Sichuan	103.21	31.42	14	42	3.87	0.08	0.616	0.037	0.053
SJ	Shuajingsi, Sichuan	102.61	32.02	32	58	4.36	0.00	0.680	0.007	0.385
LD	Luding, Sichuan	102.27	29.80	34	44	3.61	0.00	0.586	−0.034	0.313
MK	Ma'erkang, Sichuan	102.22	31.90	29	58	4.38	0.03	0.674	0.084	0.423
KD	Kangding, Sichuan	101.96	30.03	33	56	4.03	0.04	0.603	−0.04	0.539
YJ	Yajiang, Sichuan	101.26	30.04	23	39	3.33	0.01	0.535	−0.003	0.246
BT	Batang, Sichuan	99.39	30.30	34	45	3.26	0.01	0.490	0.064	0.722
XZ	Milin, Xizang	94.25	29.22	9	32	3.13	0.00	0.449	−0.04	‐
YL	Yulongxian, Yunnan	100.28	27.20	28	32	2.69	0.00	0.418	0.103	0.577
PD	Pudacuo, Yunnan	99.94	27.90	15	23	2.46	0.00	0.336	−0.043	0.002*
HP	Hongpocun, Yunnan	99.82	27.81	32	38	2.85	0.05	0.395	0.052	0.577
DN	Deqin, Yunnan	98.91	28.45	29	30	2.34	0.00	0.303	−0.128	0.539
RS	Russia far east, Russia	131.59	43.14	10	53	5.05	0.01	0.727	−0.001	0.053
FJ	Fujiyama, Japan	138.73	35.36	15	50	4.41	0.15	0.681	0.021	0.216

Abbreviations: *A*_O_, observed allele number; *A*_r_, allele richness (based on 16 genes); *F*_IS_, fixation index; *H*_E_, expected heterozygosity; *n*, sample size; *P*_A_, private allelic richness.

* *p* < 0.05.

### 2.2 DNA extraction and microsatellite genotyping

Total genomic DNA was extracted from 20 mg of dried leaf tissue from each individual white birch and purified using a Plant Genomic DNA Extraction Kit (Tiangen) following the manufacturer's instructions. We tried 32 cpDNA primers, all of which were invariant along the species range. Ten pairs of microsatellite primers developed for *B. platyphylla* var. *japonica*, *Betula maximowicziana, Betula pendula* (Kulju, Pekkinen, & Varvio, 2004; Tsuda, Ueno, Ide, & Tsumura, 2008a; Tsuda, Ueno, Ranta, et al., 2008b; Wu & Hogetsu, 2002) were organized into three multiplex PCR groups (Table S1) and used to amplify all of the individuals. PCR amplifications were performed with a Veriti 96‐Well Thermal Cycler (Applied Biosystems) using 5 µL reactions containing 20 ng of genomic DNA, 1 × QIAGEN Type‐it Microsatellite PCR Kit (QIAGEN), and various concentrations of primers labeled with fluorescent dye, FAM, ROX, TET, or HEX (Sangon) (Table S1). PCR cycles consisted of an initial denaturing step of 5 min at 95°C, followed by 14 cycles of 95°C for 30 s, touchdown annealing from 65°C to 58.5°C (Δ = 0.5°C) for 3 min and 72°C for 30 s, followed by 15 cycles of 95°C for 30 s, 58°C for 3 min, and 72°C for 30 s, and a final elongation step of 30 min at 60°C. PCR products were analyzed in an ABI 3730xl capillary sequencer (Applied Biosystems) with LIZ 500 as an internal standard. Fragment sizes were assessed using GeneMapper v.4.0 (Applied Biosystems) and checked manually twice to reduce scoring error.

### 2.3 Microsatellite data analysis

#### 2.3.1 Genetic diversity analysis

The presence of null alleles was checked with MICROCHECKER v. 2.2.3 (Van Oosterhout, Hutchinson, Wills, & Shipley, 2004). Genotypic linkage disequilibrium was tested for all locus pairs in each population by randomization, and the obtained *P*‐values were adjusted applying a sequential Bonferroni correction (Rice, 1989) to avoid false positives, using FSTAT v. 2.9.3 (Goudet, 2001). For each microsatellite locus, genetic diversity was assessed by calculating the observed number of alleles (*A*_O_), the observed heterozygosity (*H*_O_), the genetic diversity within populations (*H*_S_), and the total gene diversity (*H*_T_). For each population, descriptive statistics such as the observed number of alleles (*A*_O_), the expected heterozygosity (*H*_E_), and the fixation index (*F*_IS_) were calculated across all nSSR loci. These statistics were estimated using FSTAT v. 2.9.3 (Goudet, 2001). Allele richness (*A*_r_) and private allelic richness (*P*_A_) for each population were calculated by rarifying to 16 gene copies using HP‐RARE v. 1.1 (Kalinowski, 2005). The significance of deviations from Hardy–Weinberg equilibrium (HWE), given by deviation of fixation index (*F*_IS_) from zero, was tested by randomization using FSTAT v. 2.9.3 (Goudet, 2001). To determine whether genetic variation within populations was correlated with geographical gradients, Pearson correlations between statistics of variation (*A*_O,_ *A*_r_, *P*_A,_ and *H*_E_) and geographical ordinates (latitude) for each population were analyzed in the package SPSS v. 20.0.

#### 2.3.2 Genetic differentiation and genetic structure analysis

We used *θ* (*F*_ST_) (Weir & Cockerham, 1984) and the standardized genetic differentiation *G*'_ST _(Hedrick, 2005) to evaluate the level of genetic differentiation among all populations across ten loci with FSTAT v. 2.9.3 (Goudet, 2001). Genetic structure was investigated using different approaches. Distinct gene pools were inferred using a Bayesian clustering method implemented in STRUCTURE v.2.3.4 (Falush, Stephens, & Pritchard, 2003; Pritchard, Stephens, & Donnelly, 2000). A total of 20 independent simulations were run for each *K* (=1–8) with 50,000 burn‐in steps followed by 500,000 MCMC (Monte Carlo Markov chain) steps using the admixture model with correlated allele frequencies. STRUCTURE HARVESTER (Earl & Vonholdt, 2012) was employed to calculate the probability of the data for each *K* and to calculate Δ*K* according to the method described by Evanno, Regnaut, and Goudet (2005). The main pipeline of Clumpak (Kopelman, Mayzel, Jakobsson, Rosenberg, & Mayrose, 2015) was then executed for the summation and graphical representation of the results previously obtained by STRUCTURE. Principal coordinates analysis (PCoA) was also conducted on the microsatellite data using GENALEX v.6.502 (Peakall & Smouse, 2012), via a distance matrix with data standardization.

A Mantel test (linear codominant genetic distance vs. geographical distance; 999 permutations) was conducted in GENALEX v.6.502 (Peakall & Smouse, 2012) to determine whether genetic distance matrices were correlated with geographical distance. The same test was carried out separately for each cluster defined by STRUCTURE. Subsequently, we compared genetic diversity and population differentiation among the clusters using FSTAT v.2.9.3 (Goudet, 2001). AMOVA (Excoffier, Smouse, & Quattro, 1992) was used to partition total molecular variance at different levels in ARLEQUIN v.3.5.2.2 (Excoffier & Lischer, 2010). Groups for AMOVA were defined according to the result of STRUCTURE. Significance was obtained by nonparametric permutation using 10,000 replicates.

### 2.4 Historical demography

#### 2.4.1 Gene flow

Historical rates and direction of gene flow between the five STRUCTURE groups were estimated using the Bayesian inference in MIGRATE‐N v.3.6.11 (Beerli, 2006). The software was used to estimate mutation‐scaled effective population sizes (*θ* = 4*N*_e_ *μ*; *N*_e_, effective population size; *μ*, mutation rate per site per generation) and migration rates (*M* = *m*/*μ*; *m*, immigration rate per generation), using coalescence. The starting values for *θ* and *M* were estimated from *F*_ST,_ and proposal distribution was Metropolis–Hastings sampling. Under a Brownian motion mutation model, 1 long chain and three concurrent chains (3,000,000 trees) were run with an initial burn‐in of 10,000 trees. We used a static heating scheme at four temperatures (1, 1.5, 3, and 10^4^) to efficiently search the genealogy space.

#### 2.4.2 Bottlenecks

We used the program BOTTLENECK v. 1.2.2 (Piry, Luikart, & Cornuet, 1999) to identify populations that had recently experienced a severe reduction in effective population size (Cornuet & Luikart, 1996). The two‐phase mutation model (TPM) was used with 95% single‐step mutations and 5% multiple‐step mutations (multiple‐step variance = 12) to detect populations with a sample size ≥ 10 to increase the detection power (Piry et al., 1999).

#### 2.4.3 Approximate bayesian computation

**Figure 3:**
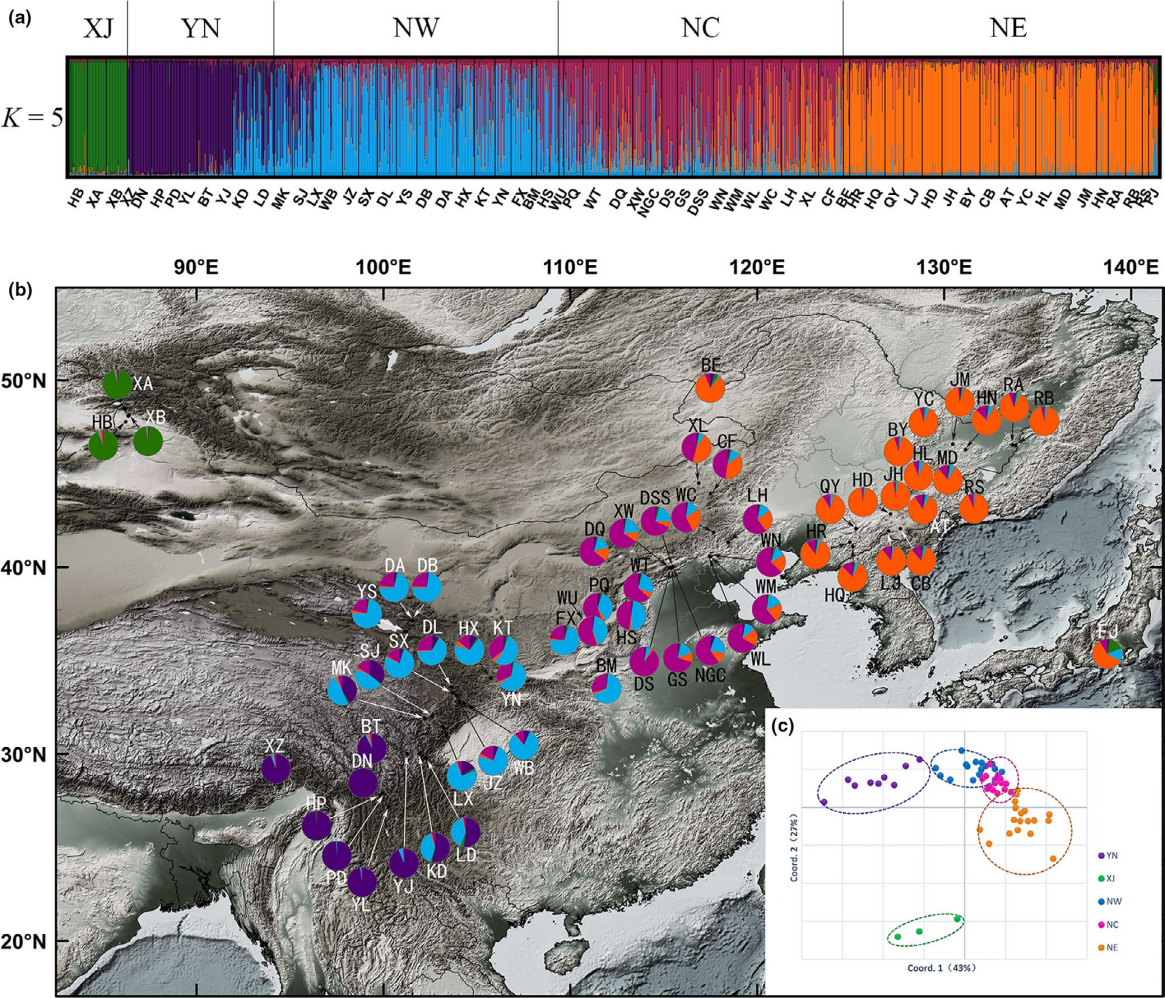
(a) Proportion of genetic clusters at *K* = 5 for each of the 1756 white birch individuals. The five clusters are delimited by black lines along the top of the plot. XJ, Xinjiang cluster; YN, Yunnan cluster; NW, northwest China cluster; NC, north China cluster; NE, northeast China cluster. (b) Geographical distribution of the five genetic clusters and composition of the genetic cluster in each population. Population codes are identified in Table 1. (c) Principal coordinates analysis (PCoA) of the relationships between sampled populations of *Betula platyphylla*

**Figure 4:**
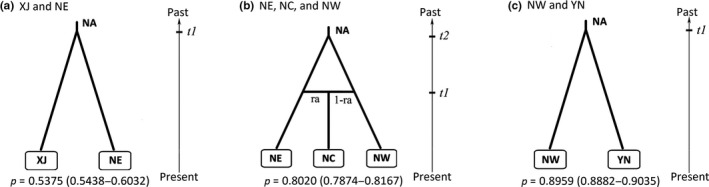
Graphical representation of the optimal scenarios of the three steps (a‐c) tested by approximate Bayesian computation. NA refers to effective size of putative ancestral, and t1 and t2 to divergence times (prior settings of population parameters are listed in Table S2). Posterior probabilities (*P*) of the scenarios and 95% confidence intervals of *P* (in brackets) computed using a logistic regression estimate are given under each scenario

To understand how the modern genetic structure of *B. platyphylla*, as shown in the STRUCTURE analysis and PCoA, was generated, we estimated divergence times, admixture, and changes in population size among different lineages (i.e., NE, NC, NW, YN, XJ, see Figure 3, later) using DIYABC v.2.1.0 (Cornuet et al., 2014; Cornuet, Ravigne, & Estoup, 2010). Since increasing the complexity of the model can lead to poor estimation of parameters (Bertorelle, Benazzo, & Mona, 2010), we tested the origins of the five lineages obtained by STRUCTURE analysis in three steps to simplify the scenarios. The first step was used to check out the relationship between NE and XJ, which are at the same latitude range in the north and share the same gene pool at *K* = 2 (see the result of STRUCTURE, Figure S2); the second step was used to explore the evolutionary history of three adjacent lineages: NE, NC, and NW; the third step was used to test the origin of YN in the southwest corner. In total, we tested 14 alternative scenarios which were described by times in generations (t1, ta, t2, t3), effective population size of putative ancestral, standing and founding populations (NA, X, Xa) (Figure 4). We gave each scenario a uniform prior probability, and specific values of prior distributions are presented in Table S2. The observed and simulated genetic data sets were summarized using the following summary statistics: the mean number of alleles and the mean genic diversity for each lineage, and the mean number of alleles, the mean genic diversity, the *F*_ST _(Weir & Cockerham, 1984), and the classification index (Rannala & Mountain, 1997) for each of the lineage pairs. One million simulations were run for each scenario.

After all the simulations had been run, a PCA (principal component analysis) was applied to pre‐evaluate the combination between observed data sets and simulated data sets and to ensure that the setting of reference tables was appropriate. Subsequently, we used 1% of the simulated data sets closest to the observed data to estimate the relative posterior probabilities for each scenario via logistic regression (Cornuet et al., 2008). Posterior parameter distributions were estimated from 1% of the closest data sets simulated according to the most likely scenario for each step. The time parameters were converted into years by multiplying generation time, which was set to 30 years for *B. platyphylla* (Osumi, 2005; Tsuda et al., 2017). Finally, we performed a model checking analysis with PCA to assess the goodness of fit of the chosen scenario.

### 2.5 Present and past distribution modeling

We used ecological niche models to identify the potential location of *B. platyphylla* at different time periods (the last glacial maximum, the mid‐Holocene, and the current time), in order to identify regions of environmental stability where the species may have persisted overtime (the possible refugia). By identifying historical shifts in suitable habitats, which expanded and contracted during glacial and interglacial periods, we also tried to infer the possible recolonization pattern (Alvarado‐Serrano & Knowles, 2014).

In addition to our sample locations (Table 1), the distribution records of *B. platyphylla* sourced from the Chinese Virtual Herbarium (http://www.cvh.ac.cn) and the Global Biodiversity Information Facility (http://www.gbif.org) were also included. To reduce the effects of spatial autocorrelation in climate variables, we removed duplicate records within 24 km of one another (12 arc‐min), leaving a total of 155 presence records for use from throughout the species range. Considering the dispersal ability of *B. platyphylla*, we buffered the area around the occurrence points by 1,000 km and selected the background points within this range. The ecological layers for the current time, the mid‐Holocene (MIH, *c*. 6 kyr BP), and the last glacial maximum (LGM, *c*. 21 kyr BP) were downloaded at 2.5’ spatial resolution. For MIH and LGM prediction, we used both CCSM4 and MIROC‐ESM database. We downloaded 37 environmental variables from WorldClim (http://www.worldclim.org) (Hijmans, Cameron, Parra, Jones, & Jarvis, 2005) and ENVIREM (http://envirem.github.io/#downloads) (Title & Bemmels, 2018); selected variables correlated *r* < 0.7 (Table S4) and retained variables with VIF (variance inflation factor) < 3 (Table S5). Temperature annual range, mean temperature of wettest quarter, precipitation seasonality, precipitation of coldest quarter, mean monthly potential evapotranspiration of the driest quarter, climatic moisture index and terrain roughness index were used in the models.

To infer the potential species distribution, we used three algorithms in BIOMOD: (a) maximum entropy; (b) generalized boosting models; (c) classification tree analysis (Thuiller, 2003; Thuiller, Lafourcade, Engler, & Araujo, 2009). For each algorithm, we used 75%–25% of occurrence points for training/testing. We evaluated the models using the TSS value (acceptable models present TSS values > 0.7 (Allouche, Tsoar, & Kadmon, 2006). We repeated this procedure 15 times for each algorithm then used the ensemble approach (Araujo & New, 2007) to create a consensus map for each climate scenario. After transforming each map in binary (the TSS threshold), we compared suitability difference between temporal scenarios (pairwise) to infer the expansion and contraction of *B. platyphylla* overtime.

## 3 RESULTS

### 3.1 Genetic diversity of the nSSR loci

The frequency of null alleles at each of the ten loci was low (only bet 48 *r* = 0.016, the others *r* = 0), and the genotypes had been adjusted. No significant genotypic linkage disequilibrium was detected between pairs of loci (*p* > 0.05). The 1756 sampled individuals of *B. platyphylla* revealed 180 alleles across the ten nSSR loci. For characteristics of individual microsatellite loci, see Table S1. The genetic diversity was highly variable among populations, with *A*_O_ ranging from 23 to 88, *A*_r_ from 2.34 to 6.03, *P*_A_ from 0 to 0.33, *H*_E_ from 0.303 to 0.795, and *F*_IS_ from −0.16 to 0.137 (Table 1). No significant deviation from HWE was detected. A significant increase in the observed number of alleles (*r* = 0.783, *p* < 0.0001), allelic richness (*r* = 0.849, *p* < 0.0001), private allelic richness (*r* = 0.485, *p* < 0.0001), and expected heterozygosity (*r* = 0.765, *p* < 0.0001) was found with increasing latitude (Figure 2).

### 3.2 Genetic differentiation and genetic structure

The multilocus *F*_ST_ was 0.107 (range 0.076–0.131, Table S1), with 99% CIs ranging from 0.091 to 0.121. The overall value of *F*_ST_ corresponded to a standardized genetic differentiation (*G*'_ST_) (Hedrick, 2005) of 0.111 (range 0.077–0.137), suggesting that the level of population differentiation was rather low.

Using STRUCTURE, the estimated logarithm of probability of the data, Ln P(*K*), increased progressively from *K* = 1 to *K* = 5 (except *K* = 4) and then plateaued (Figure S1a). The highest Δ*K* was observed at *K* = 5 but also significant at *K* = 2 (Figure S1b). At *K* = 5, 63 *B. platyphylla* populations were divided into the following geographical groups: XJ that only consisted of three populations from Xinjiang province: YN that consisted of populations from Yunnan province at the Hengduan mountains; NW that consisted of populations from northwest China plus 4 populations from north Sichuan; NC consisting of populations from north China; and NE consisting of populations from northeast China plus the two populations from Russia and Japan, respectively. A couple of populations from NW and NC showed signs of genetic admixture. Geographical distributions and other details of the 5 groups can be found in Figure 3b. A similar genetic structure was detected by the PCoA based on Nei's standard genetic distance (Figure 3c).

Mantel tests revealed a significant correlation between genetic differentiation and geographical distance across the entire range (*r* = 0.406, *p* = 0.001). However, the tests within the NE and NC lineage were not significant (NC, *r* = 0.011, *p* = 0.124; NE, *r* = 0.008; *p* = 0.262), suggesting that geographical distance did not impact the genetic differentiation within these two lineages. Measures of genetic diversity (*A*_r_, *H*_O_, *H*_S_, *F*_IS_) were significantly different among the five groups (e.g., the value of *A*_r_, XJ > NE = NC > NW> YN). The YN regional cluster of populations attained lower values of *A*_r_, *H*_O_, *H*_S,_ and *F*_IS _than the other population clusters, and the *F*_ST_ among populations in YN cluster was much higher than the others (Table 2). AMOVA results revealed significant population genetic differentiation at the range‐wide scale (*F*_ST_ = 0.059, *p* < 0.001), with 4.58% (*F*_CT = _0.046) of the variation partitioned among the five groups, and 2.29% (*F*_SC = _0.024) among populations within groups (Table 3).

**Table 2:** Differences between the five groups of *Betula platyphylla* in allele richness (*A*_r_), observed heterozygosity (*H*_O_), gene diversity (*H*_S_), fixation index (*F*_IS_), and among‐population differentiation (*F*_ST_) investigated with 5,000 permutation tests

Groups	*A*_r_	*H*_O_	*H*_S_	*F*_IS_	*F*_ST_
XJ	5.839	0.698	0.781	0.106	0.029
NE	4.81	0.669	0.698	0.041	0.033
NC	4.81	0.694	0.712	0.025	0.018
NW	4.319	0.651	0.673	0.033	0.049
YN	3.061	0.472	0.473	0.001	0.127
*p*‐value	0.0002**	0.0022**	0.0012**	0.0226*	0.194^ns^

Abbreviation: ns, not significant.

* *p* < 0.05.

** *p* < 0.01.

**Table 3:** The analysis of molecular variance (AMOVA) for nSSR data among five groups (XJ, YN, NW, NC, and NE) and all populations of *Betula platyphylla*

Source of variation	Sum of squares	Variation components	Percentage of variance (%)	*F*‐statistics
Five groups				
Among groups	555.51	0.196	4.58	*F*_CT = _0.046**
Among populations within groups	541.00	0.098	2.29	*F*_SC = _0.024**
Within populations	13,572.07	3.896	93.13	*F*_ST = _0.069**
Total populations				
Among populations	1,107.46	0.253	5.86	*F*_ST = _0.059**
Within populations	13,702.47	4.064	94.13	

** *p* < 0.01 (10,100 permutations).

### 3.3 Inference of demographic history

#### 3.3.1 Migration and bottlenecks

The results based on migration inference showed that there was a certain level of gene flow between the five distribution regions of *B. platyphylla* (*Mθ*_i_ = 0.198–34.578). The minimum gene flow was YN to XJ, 0.198 (0–0.599), and the maximum gene flow was NE to NC, 34.578 (22.551–37.800). Gene flow between XJ and the other four regions was generally weak, consistent with the remote geographical location of Xinjiang populations. The other four regions were adjacent in turn. From south to north, the gene flow between adjacent regions increased gradually, and there was clear asymmetric gene flow between NE and NC (NE → NC, 34.578 (22.551–37.800); NC → NE, 20.244 (17.322–22.733)), suggesting that NC obtained more pollen or seeds from NE (Table 4). Under the TPM model, three populations were identified to have experienced bottlenecks recently: JH in northeast China, DS in north China, and PD in southwest China (Table 1).

**Table 4:** Historical gene flow and 95% confidence intervals (CI) (in parentheses) between five *Betula platyphylla* geographical regions using MIGRATE

Regions	*θ* **_i_**	*Mθ* **_i_** = 4*Nm*				
		XJ → i	YN → i	NW → i	NC → i	NE → i
XJ	0.015 (0.010–0.017)	–	0.198 (0–0.599)	1.571 (0.684–2.383)	2.760 (1.561–3.845)	2.818 (1.663–3.718)
YN	0.027 (0.021–0.030)	2.061 (1.044–2.973)	–	**9.361 (6.734–11.260)**	8.290 (5.509–10.512)	8.379 (6.094–9.843)
NW	0.088 (0.084–0.091)	3.065 (1.227–5.042)	**9.078 (6.521–15.735)**	–	**22.447 (19.171–26.671)**	24.316 (21.067–27.764)
NC	0.098 (0.093–0.100)	4.009 (1.425–6.467)	12.351 (9.230–15.000)	**22.194 (18.648–25.733)**	–	**34.578 (22.551–37.800)**
NE	0.098 (0.096–0.100)	2.198 (0.255–4.200)	7.710 (5.158–10.200)	17.028 (13.947–20.667)	**20.244 (17.322–22.733)**	–

Note

The data in bold are the gene flow between adjacent regions. Directionality of gene flow is read from geographical regions on top being the source populations, whereas geographical units to the left are the recipient populations. *θ*_i_, effective population sizes; *M*, migration rates; *Mθ*_i_, the number of immigrants per generation.

#### 3.3.2 DIYABC

The results of the first step showed that, before 86, 600 years BP (95% CI: 21, 540–253, 500), if assuming a generation time of 30 years, populations from XJ and NE had a common ancestor that diverged into two different lineages (Figure 4a, Table 5 step 1, scenario 1, *p* = 0.574 (0.544–0.603)). The median values of the effective population sizes of XJ and NE were 6,870, 7,520 respectively. According to the results of the second step, we reconstructed the evolutionary history of the three adjacent lineages NE, NC, and NW, NE and NW were separated at 22, 170 years BP (95% CI: 5,340–96,000), and the central lineage (NC) was generated by admixture of NE and NW at 1902 years BP (95% CI: 510–6,270) (Figure 4b, Table 5 step 2, scenario 4, *p* = 0.802 (0.787–0.817)). The estimated admixture coefficient suggested that 35.6% of the nuclear genome of the NC lineage in the initial population was derived from NE, and 64.4% came from NW. The median effective population sizes of NE, NC, and NW were 9,000, 6,400, and 4,240, respectively. When inferring the origin of populations growing in the area at southwest corner (YN and NW), divergence between YN and NW was estimated to have happened around 27, 510 years BP (95% CI: 6,330–111,300) as implied by scenario 1 (*p* = 0.896, 0.888–0.904, Figure 4c, Table 5 step 3). The median population sizes of YN and NW were 2,530 and 8,540, respectively. More posterior probability distribution data could be found in Table S3, and the model checking results for the best scenario in each step were shown in Figure S3.

**Table 5:** Description of the 14 scenarios in ABC analysis for *Betula platyphylla* and posterior probability of each scenario and its 95% confidence intervals (in parentheses) based on the logistic estimate by DIYABC

Scenario	Description	Posterior probability
Step 1 XJ and NE		
Scenario 1	Both refugia	*p* = 0.5375 (0.5438–0.6032)
Scenario 2	Expansion from XJ to NE	*p *= 0.3003 (0.2712–0.3295)
Scenario 3	Expansion from NE to XJ	*p *= 0.1262 (0.1088–0.1436)
Step 2 NE, NC and NW		
Scenario 1	Expansion from NE to NC	*p *= 0.0028 (0.0000–0.0620)
Scenario 2	Expansion from NW to NC	*p *= 0.0094 (0.0000–0.0683)
Scenario 3	All refugia	*p *= 0.1538 (0.0931–0.2144)
Scenario 4	Admixture in NC from NE and NW	*p *= 0.8020 (0.7874–0.8167)
Scenario 5	Expansion from NE to NC and then from NC to NW	*p *= 0.0030 (0.0000–0.0621)
Scenario 6	Expansion from NC to NE and NW	*p *= 0.0157 (0.0000–0.0746)
Scenario 7	Expansion from NW to NC and then from NC to NE	*p *= 0.0005 (0.0000–0.0598)
Scenario 8	Expansion from NC to NW	*p *= 0.0129 (0.0000–0.0717)
Step 3 NW and YN		
Scenario 1	Both refugia	*p *= 0.8959 (0.8882–0.9035)
Scenario 2	Expansion from YN to NW	*p *= 0.0921 (0.0847–0.0995)
Scenario 3	Expansion from NW to YN	*p *= 0.0120 (0.0103–0.0137)

### 3.4 Present and past ecological niche models

**Figure 5:**
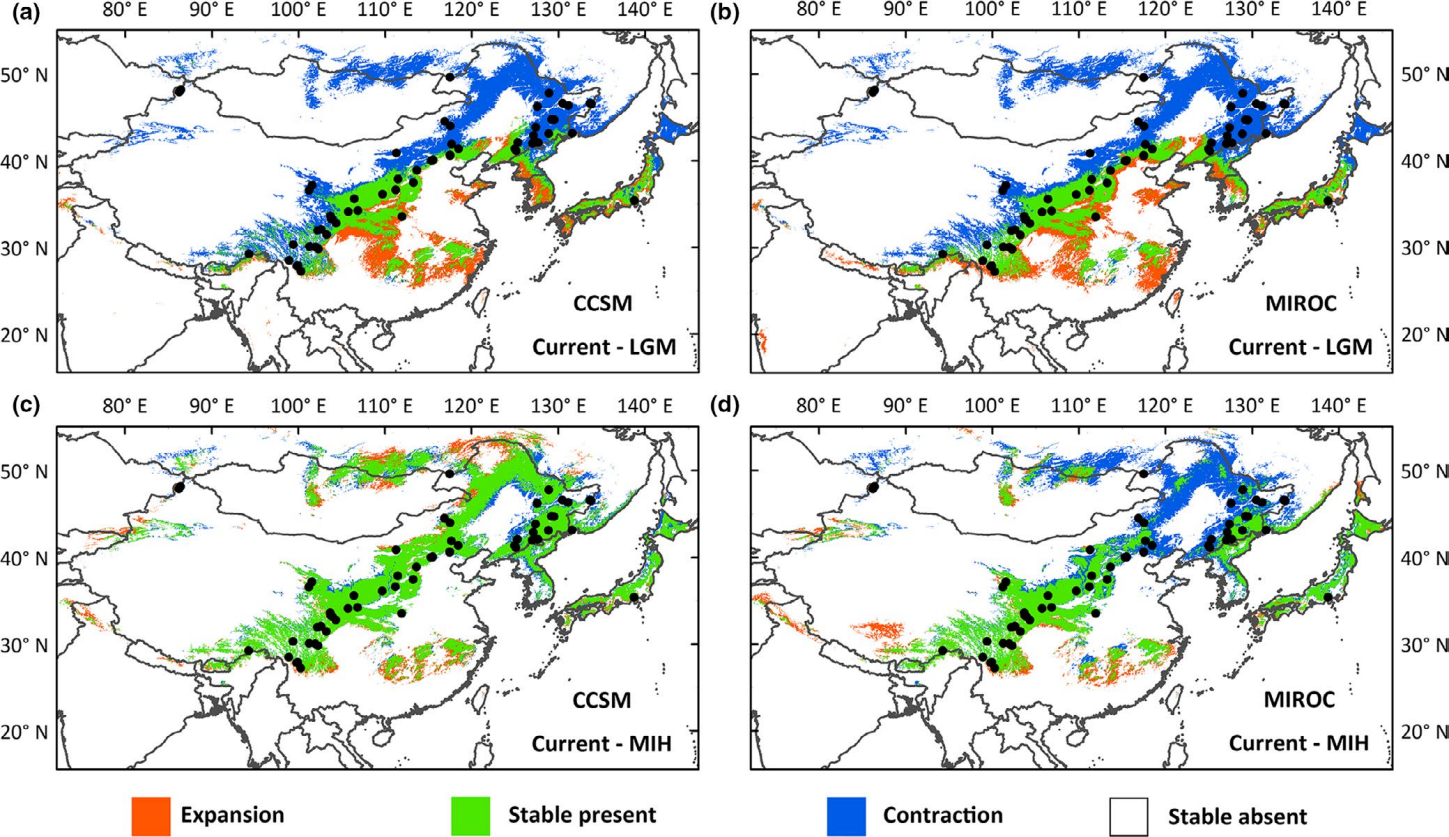
Predicted distribution area under MIH and LGM conditions as compared to current predictions. Black dots are our sample locations

As the weighted average value of TSS is 0.798, we assumed that the models generated reliable predictions. The species showed contractions (Figure 5 color blue) during historical periods compared to under the current conditions, suggesting recent expansion. Although suitable distribution areas were predicted along the southeast coast, *B. platyphylla* was not distributed there in reality due to historical dispersal events or other possible reasons. In the LGM, *B. platyphylla* showed slight shifts in its centroid of distribution toward the southeast both in the CCSM model and the MIROC model. Although there is a large area of stable present distributions (Figure 5 color green), white birch populations experienced losses of suitable habitat in their northern locations, with the distribution compressed to around 45°N, while shrinking a little in the southeastern region (around 30°N). The CCSM model for the MIH showed similar distribution with current conditions and even slight expansion of suitable areas above 45°N for the northern part, whereas for the MIROC model, suitable habitats in the same region were predicted to be more fragmented (Figure 5).

## 4 DISCUSSION

Our analysis based on nSSRs found an increase in genetic diversity with latitude (Figure 2) and relatively low genetic differentiation among populations of *B. platyphylla*. The result based on genetic structure suggested that Asian white birch populations could be divided into five groups, XJ, NE, NC, NW, and YN (Figure 3). The southwestern group YN harbored significantly lower genetic diversity (Table 2). Different levels of multidirectional gene flow resulted in admixture among different groups (Table 4). The approximate Bayesian computation (ABC) analysis showed that populations in the north and the south diverged into different lineages, respectively, as Quaternary climate got cold and dry, and admixture occurred in north China, when conditions ameliorated (Figure 4). Ecological niche modeling (Figure 5) indicated that the species probably took refuge in both northern and southern parts of East Asia. Moreover, the distribution of northern populations was relatively variable, whereas southern populations seemed to stay in situ.

### 4.1 Persistence both in the north and in the south without long‐distance migration

Long‐distance migration in the past left traces of the genetic patterns of species, and in most cases, newly established marginal populations showed lower genetic diversity than long‐standing populations (Bialozyt, Ziegenhagen, & Petit, 2006; Ohsawa & Ide, 2011; Waters, Fraser, & Hewitt, 2013). Yet the genetic pattern of some species, such as walnuts (*Juglans* spp.), is exceptions, because of limited dispersal mechanisms (Wang et al., 2016). For species with the capacity for long‐distance dispersal, such as birches, if the species had experienced long‐distance migration from low to high latitudes, genetic diversity should decrease with latitude increasing. However, our nSSR data showed that genetic diversity of white birch increased with latitude (Figure 2), and genetic parameters, such as *A*_r_ and *H*_O_, were higher in northern (XJ, NE, and NC) than in southern groups (NW, YN) (Table 2). This pattern contradicts the southern refuge model, indicating that large‐scale southward retreat followed by northward recolonization does not apply to widespread Asian white birch (Harrison et al., 2001).

Genetic diversity gradually decreased from north to south, implying that white birch possibly had experienced a range expansion from north to south (Slatkin & Excoffier, 2012). However, at the LGM, the southern groups persisted in the mountainous areas surrounding the southeastern edge of the Qinghai–Tibetan Plateau (QTP), and the northern groups contracted to around 45°*N* (Figure 5). Furthermore, ABC modeling suggested that long‐distance expansion did not exist (Table 5, step 2 scenario 5, 7). The most likely scenarios suggested that XJ, NE, NW, and YN had undergone relatively independent evolutional histories (except NC in north China showed signs of admixture), and the divergence occurred during the LGM or earlier glacial periods (Figure 4), providing support for intraspecific differentiation driven by Quaternary climatic fluctuations. This combined evidence jointly suggest that *B. platyphylla* survive the glacial periods in the north and the south separately and the present distribution of the species originated from local recolonization from multiple refugia, rather than long‐distance migration.

Persistence in both the northern and the southern region sustaining multiple glacial refugia during historical climatic changes has also been found in the study of *Lindera obtusiloba*, although the divergence time between the two regions was dated to the Pliocene (Ye et al., 2017). There exist several drivers of genetic differentiation in plants, such as isolation by dispersal limitation, colonization, and/or adaption (Orsini, Vanoverbeke, Swillen, Mergeay, & De Meester, 2013). Maybe more than one driver have affected the genetic pattern of *B. platyphylla*. According to the ecological niche models, compared to the current relatively continuous habitat of white birch, the northern and southern regions were isolated and had smaller distribution ranges in history (Figure 5). Therefore, isolation and local spread may have played a major role in the process of historical differentiation of *B. platyphylla*. However, the degree and role of adaption would still need to be detected by combining neutral and non‐neutral markers in the future (Orsini et al., 2013).

Notably, NE is located in an area above 40°N, including the Changbai Mountains, the Daxing'anling Mountain range, and the Xiaoxing'anling mountain range, adjacent to Russian Far East. Suitable habitat was not predicted by our LGM distribution model in this region, except the Changbai Mountains (Figure 5). This seems to be inconsistent with the fact that large areas of the northeastern part of China are currently covered with white birch. Besides, higher genetic diversity and private alleles, which were known as characteristics of refugia, were observed at higher altitudes in NE (Figure 2 and Table 1) (Bennett & Provan, 2008; Maggs et al., 2008). Moreover, based on a recent study conducted by Tsuda et al.(2017), *B. platyphylla* was likely to persist further north than initially thought during the glacial–interglacial cycles. Therefore, we believe there are two possible scenarios, the first scenario is that white birch might have survived the LGM in northeastern China or even further north, considering ecological niche models might not detect the effects of microclimate on distributions (Gavin et al., 2014); the second scenario is that the LGM contraction was real and northern populations expanded northward during the MIH (Figure 5c) as predicted by the CCSM model, interspecies hybridization at higher latitude (Tsuda et al., 2017) brought more genetic variation and white birch migrated southward after the MIH. We preferred the second scenario based on our known evidence.

In summary, our results indicated that *B. platyphylla* in East Asia remained almost stable over long periods of time and sustained multiple glacial refugia in the north and the south. Northern refugia were probably located in the northwest of Xinjiang (the Altay Mountains.), the north China, the northeast China, and adjacent regions (Russian Far East), and the southern refugia in northeastern (the Qilian Mountains) and southeastern (the Hengduan Mountains) edge of Qinghai–Tibetan Plateau. This scenario best explains the phylogeographical pattern of *B. platyphylla* and conforms with the fossil evidence that suggests that *Betula* had four distributional centers in eastern continental Asia (Cao et al., 2015). Given the scope of our research, the presumed refugia were in broad regions. More detailed local studies would be needed to identify specific locations of glacial refugia or microrefugia (Mee, Moore, & Linder, 2014; Zeng et al., 2015).

### 4.2 Postglacial expansion in multiple directions

Gene flow can reach spatial scales larger than distribution shifts under changing climate and genetic interactions between contrasting environments allowed forest trees to adapt to rapid climate change (Kremer et al., 2012). The scenario that *B. platyphylla* could maintain a wide distribution through time may be related to the capacity for long dispersal distances through pollen and seeds, as well as extensive historical gene flow. The postglacial migration patterns of Asian white birch, detected by MIGRATE, were characterized by wide coverage and multiple directions, since almost all the historical gene flow among populations had a 95% confidence interval greater than 0 and there were moderate to strong bidirectional gene flow among adjacent lineages (9.078–34.578). Additionally, the value of gene flow was reduced from north to south (Table 4). Inconsistently, the result of BOTTLENECK revealed that only three isolated populations, that is, JH from NE, DS from NC, and PD from YN (Table 1), had been recently bottlenecked. However, the genetic effects of a bottleneck might be obscured via the introduction of rare alleles by high gene flow among different population groups.

STRUCTURE detected an admixture‐like genetic structure in NC lineage in north China (Figure 3). The ABC suggested that the genetic structure in NC could be better explained by admixture model due to the secondary contact by NE and NW (Figure 4b). Accordingly, NC was one of the glacial refugia for white birch, but also a contact zone where genetically distinct lineages from northeast and southwest/west met. Maybe that is why the populations there have relatively higher genetic diversity (Table 2). The admixture between NE and NW was inferred to have taken place 1902 (95% CI: 510–6270) years ago. This time is very close to the hybridization time between *B. pendula* and *B. platyphylla*, which was 1614 (95% CI: 561–4710) years ago, as estimated by Tsuda et al.(2017). The combined results confirmed that *B. platyphylla* had experienced a wide‐range expansion in the late Holocene in the northern region, causing admixture and hybridization between pedigrees and different species.

Populations, located on the Loess Plateau (PQ from NC, FX, HX, and KT from NW), have very low private allelic richness values, which were close or even equal 0 (Figure 3 and Table 1). The absence of private allele within these populations indicated that they were probably derived from adjacent populations through (re)colonization rather than being relicts after a founder event or genetic drift (Maggs et al., 2008). In combination with historical rates of gene flow between NC and NW (Table 4), we can boldly infer that these populations were mainly formed from the eastward postglacial expansion and recolonization of individuals from northeastern edge of Qinghai–Tibetan Plateau into once uninhabitable regions. However, the ecological niche models of different historical periods (LGM and MIH) indicated that the Loess Plateau was in the middle zone between the northern and the southern region, where remained suitable for white birch for survival. In addition, other temperate species were reported to persist on the Loess Plateau or surrounding mountains through Quaternary glaciation (Hao et al., 2017; Zong et al., 2014). The contrasting conclusions suggest that the community composition and structure of this area in the past may be very different from the current, and *B. platyphylla* may be the pioneer of colonization when there were vacant niches.

### 4.3 Genetic pattern influenced by topographic heterogeneity

Our data indicate that genetic diversity in white birch populations was not evenly distributed across the range. The values of *A*_O_, *A*_r_, *P*_A, _and *H*_E _were high in the northeast, moderate in the middle area, and relatively low in the southwest mountainous area (Figure 2). By contrast, the values of genetic differentiation of different lineages increased from north to south (Table 2). Moreover, Mantel tests and MIGRATE suggested that long‐distance hybridization may have existed among northern populations and the rate of historical gene flow decreased southwestward along the mountains (Table 4).

There are three possible factors forming different genetic characteristics in the north and the south. Firstly, the terrain difference between the south and the north is probably the most important factor. The northern populations are located in a flatter region with no large geographical barriers. The pollen of white birch can spread far by air and be collected by tall trees at similar altitudes, leading to more frequent gene flow, which improves the possibility of hybridization, slows divergence and increases genetic diversity (Havrdova et al., 2015). The southern populations, located on the eastern margin of QTP, have more complex topography. High mountains and deep valleys separate different populations and restrict pollen and seed dispersal. Long‐time isolation, restricted gene flow, and genetic drift fixed unique genetic composition, caused more inbreeding, and resulted in strong genetic differentiation and low genetic diversity. Secondly, the shorter growing season reduces differences in flowering times among northern groups and, thereby, provides more possibilities for genetic admixture (Kallio, Niemi, & Sulkinoja, 1983). Thirdly, the refugia populations in Beringia and/or admixture populations with other tree species such as *B. pendula* (Tsuda et al., 2017) might have contributed a lot to the high genetic diversity in the north.

The influence of topographic heterogeneity is also supported by the different distribution dynamics of the northern and southern populations from the LGM to the present. As we can tell from the ecological niche models, the species responded in different ways to past climatic changes. The northern populations extended to a larger area with the relatively continuous range and have more opportunities to expand and contract in response to climate changes. Whereas the southern populations are often restricted in distribution with only slight range change (Figure 5), possibly thanks to suitable habitats provided by specific microclimates in complex topographies (Birks, 2015). This discrepancy can be attributed to diverse topographic and ecological differences between these two regions (Opgenoorth et al., 2010).

The findings that genetic pattern and distribution dynamics of *B. platyphylla* exhibited an uneven pattern indicated that this species has a broad capacity to grow under various conditions and the capacity facilitates its wide distribution range. However, the genetic and distributional difference among populations should be paid more attention in relation to species conservation. After the glacial periods, migrations from separate refugia would meet in contact zones forming high genetic diversity there (Havrdova et al., 2015; Mayol et al., 2015). As we discussed above, *B. platyphylla* showed high genetic diversity in north China because of admixture. In contrast, for marginal populations, such as YN in southwest China, the limited genetic admixture caused by topography would make them more vulnerable or even disappear in the face of climate changes. We should be careful when we formulate the policy of tree species conservation and forest management, and we should pay more attention to fragmented populations with low diversity in such areas. After all, studies of genetic variation within species allow us to track their population histories and to predict future range shifts induced by the climate change, making it possible to apply proper protection activities (Jadwiszczak, 2012).

In conclusion, the use of genomic data coupled with ecological niche modeling approach allowed us to recover for the first time a detailed demographic history of *B. platyphylla*, which covers a large area of East Asia. Our study suggests that multiple LGM refugia, with different altitudes and landforms, may have allowed temperate trees to persist in both southern and northern East Asia. Postglacial range expansion occurred in different directions leading to admixture between recolonizing populations. In addition, besides historical climate change, topographic heterogeneity might also have an impact on the formation of genetic pattern and make it possible that northern and southern populations have had different expansion/contraction dynamics. These findings reveal new perspectives on our understanding of how widespread tree species responded to climatic variation.

## AUTHOR CONTRIBUTIONS

A‐R.L. and T‐Y.C. planned and designed the research. T‐Y.C. performed experiments, analyzed data, and wrote the manuscript.

## Data Availability

Genotype files used in this study were deposited at Dryad: https://doi.org/10.5061/dryad.27jp751.
