# Supplementary material for: Phylogeography and paleodistribution models of a widespread birch (Betula platyphylla Suk.) across East Asia: Multiple refugia, multidirectional expansion, and heterogeneous genetic pattern
Source: Ecol Evol. 2019 Jun 14;9(13):7792–807. doi: 10.1002/ece3.5365 (PMC6635942; doi:10.1002/ece3.5365)
Supplement: Supplementary file 1 [file ECE3-9-7792-s001.pdf]

Supplemental Information for:

Phylogeography and paleodistribution models of a widespread birch (*Betula platyphylla* Suk.) across East Asia: multiple refugia, multidirectional expansion, and heterogeneous genetic pattern

Tian-Yi Chen, An-Ru Lou*
Ministry of Education Key Laboratory for Biodiversity Science and Ecological Engineering, College of Life Sciences, Beijing Normal University, Beijing 100875, China

The following Supporting Information is available for this article:
Fig. S1 Bayesian inference of the number of clusters (*K*).
Fig. S2 Plots of posterior probabilities for individuals of white birch assigned to *K* genetic clusters from STRUCTURE analyses for *K* = 2-4.
Fig. S3 Graphical representation of 14 competing scenarios tested in three steps (a-c) by approximate Bayesian computation.
Fig. S4 Principal Component Analysis obtained by DIYABC model checking for the three optimal scenarios.
Fig. S5 Map showing white birch points used in the climate modeling

Table S1 Summary results of microsatellite data for each loci across 63 populations of white birch.
Table S2 Prior distributions of the parameters used in DIYABC.
Table S3 Parameter estimates of the most likely scenarios obtained by DIYABC.
Table S4 Correlation analysis of reserved variables
Table S5 VIFs of the remained variables
Table S6 Ecological niche Model accuracy evaluation

Notes S1 Speculations about the populations outside China.

Fig. S1 Bayesian inference of the number of clusters (*K*). *K* was estimated using (a) the posterior probability of the data given each *K* (20 replicates) (mean ± SD) and (b) the distribution of delta *K*.

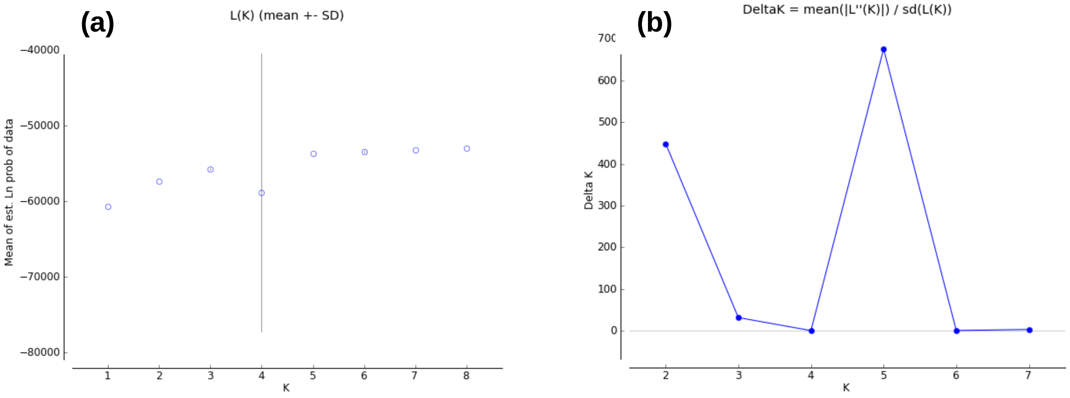

Fig. S2 Plots of posterior probabilities for individuals of *Betula platyphylla* assigned to *K* genetic clusters from STRUCTURE analyses for *K* = 2-4.

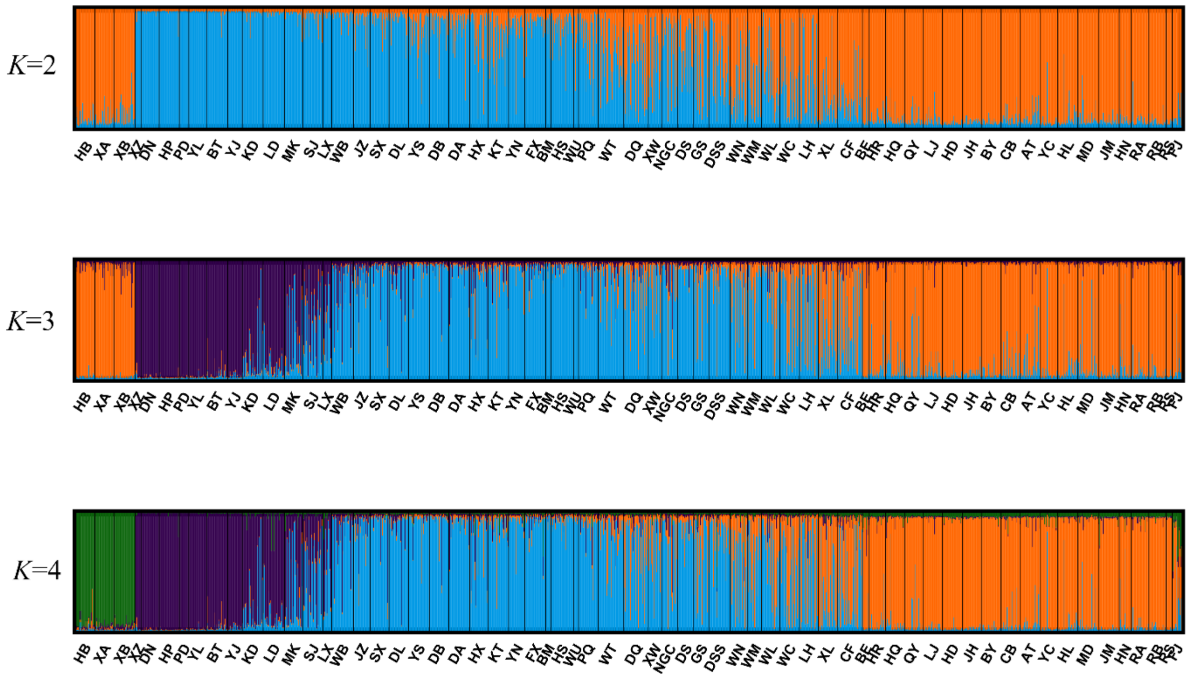

Fig.S3 Graphical representation of 14 competing scenarios tested in three steps (a-c) by approximate Bayesian computation. NA, X and Xa refer to effective sizes of putative ancestral, standing and founding populations, and t1, ta, t2 and t3 to divergence times (mutation models are mentioned within the figure and prior settings of population parameters are listed in Table S2). Posterior probabilities (*P*) of the scenarios and 95% confidence intervals of *P* (in brackets) computed using a logistic regression estimate are given under each scenario. The most probable scenario for each step is framed by black rectangle.

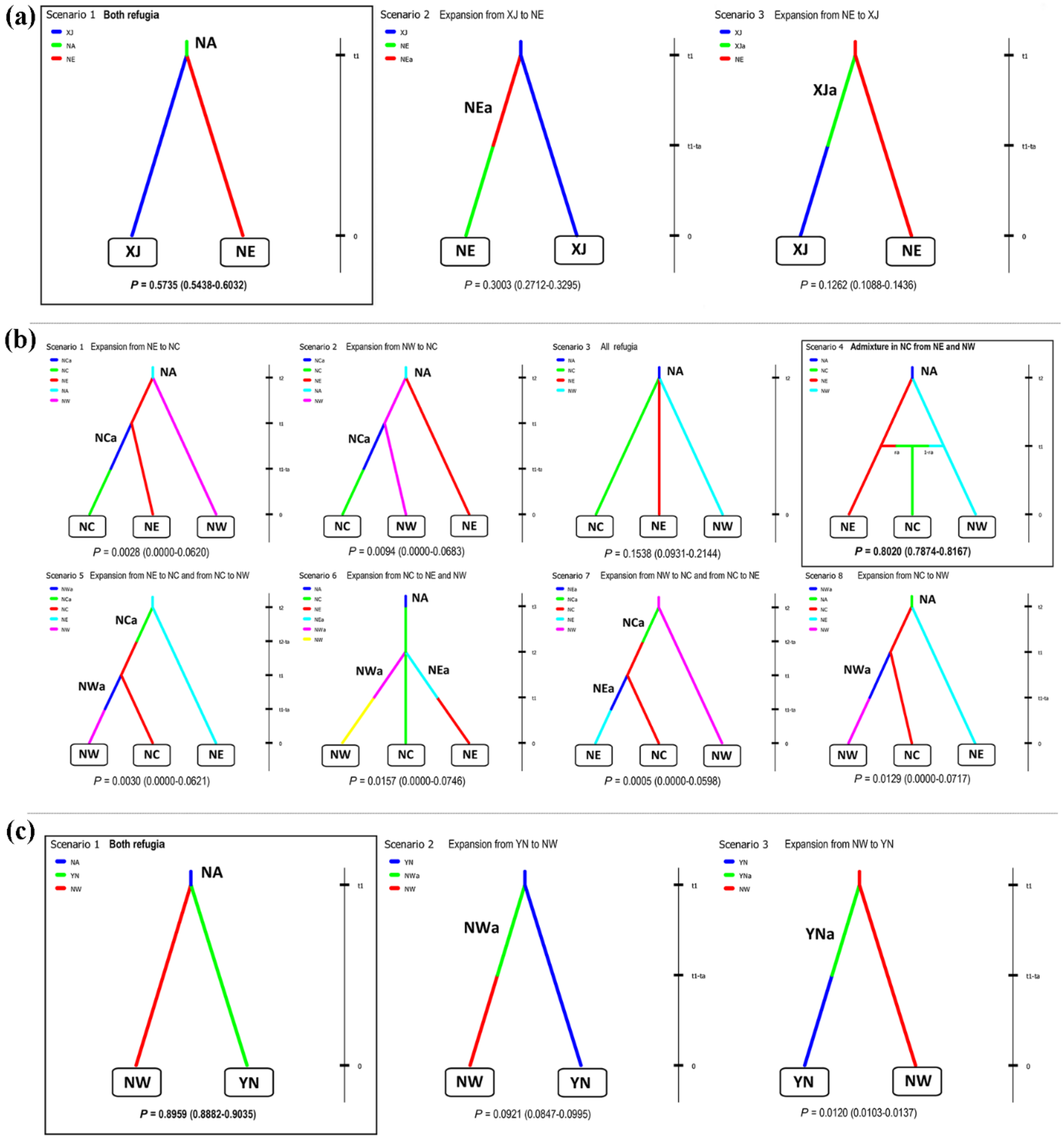

Fig.S4 Principal Component Analysis obtained by DIYABC model checking for the three optimal scenarios.

Step 1:

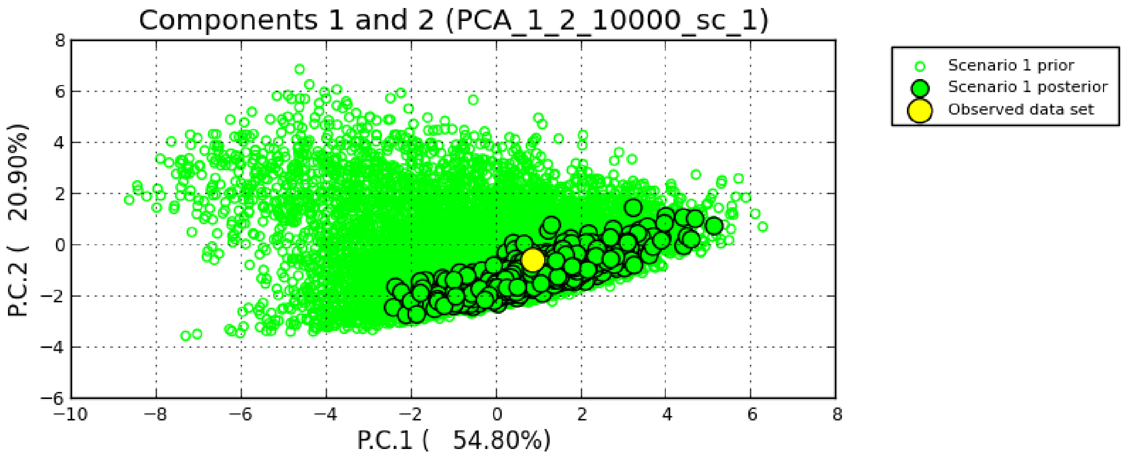

Step 2:

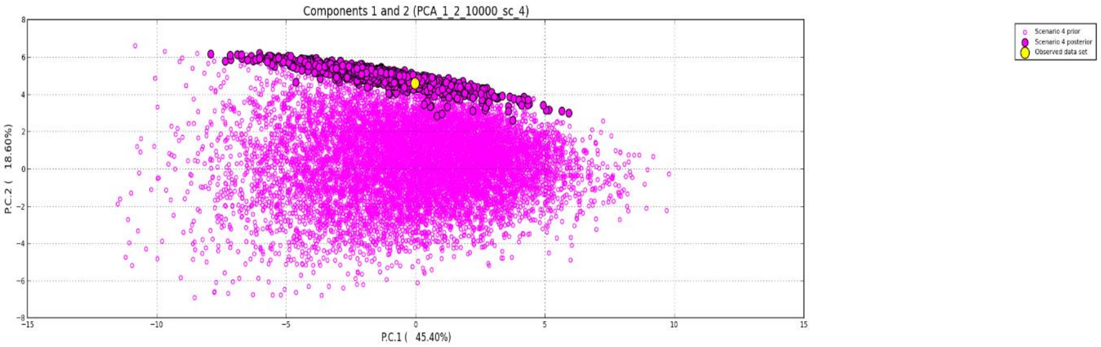

Step 3:

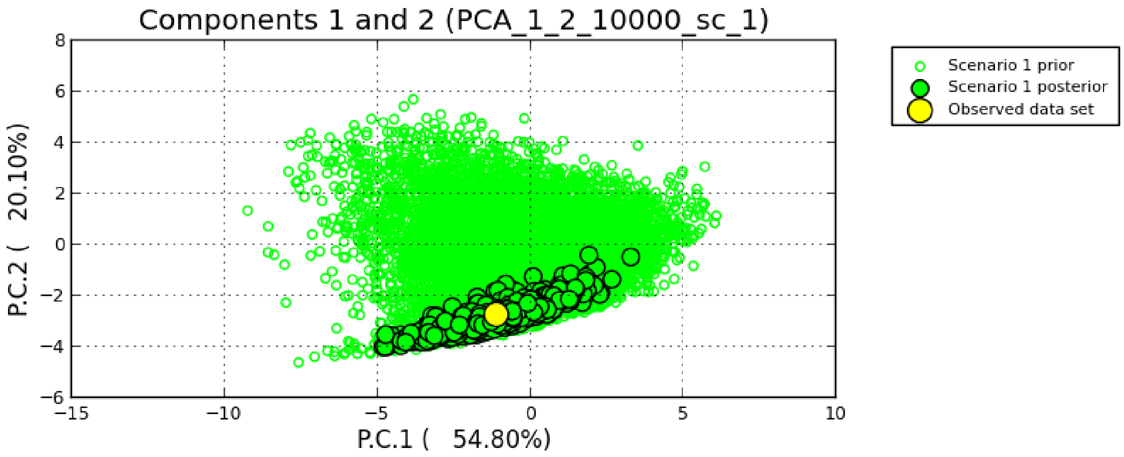

Fig. S5 Map showing white birch points used in the climate modeling

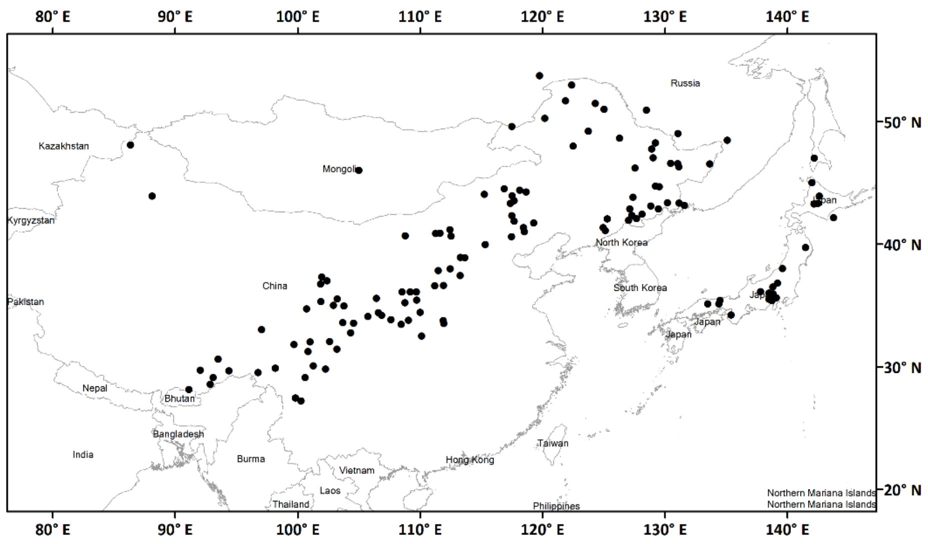

Table S1 Microsatellite loci analyzed, PCR conditions, allele size ranges, observed allele number (A_O), observed heterozygosity over all populations (H_O), gene diversity within populations (H_S), overall gene diversity (H_T), among population differentiation (F_{ST}) and the fixation index (F_{IS}) across 63 white birch populations.

Locus	Accession no.	Repeat motif	Primer sequence (5'-3')	Allele range (bp)	Fluorescent dye	concentration(μM)	Multiple group	Reference	A_O	H_O	H_S	H_T	F_{ST}	F_{IS}
Bet17	AB084478	(GT)8…(AT)4	F: GGTATTCAGACTGCTCATACC<br>R: TATCCTATGCAAGGGACGAAG	106-120	FAM	0.2	1	Wu *et al*. (2002)	7	0.612	0.656	0.731	0.099	0.06
Bet21	AB084482	T9(GT)13	F: ACGCTTTCTTGATGTCAGCC<br>R: TCACCAAGTTCCTGGTGGAT	164-234	TET	0.2	1	Wu *et al*. (2002)	32	0.764	0.76	0.86	0.112	-0.004
Bet1	CD276757	(TAT)7GAGT(TA)4	F: AGTACGTAGAAAATGACGGGGATG<br>R: GTTTAACCAGAAACTACCACGTGAACCA	199-223	ROX	0.4	1	Tsuda *et al*.(2008a)	9	0.634	0.637	0.69	0.079	0.003
Bet30	CD277217	(AC)4TTTTTCCGGG<br>TGCGTTA(CT)3G(TC)6	F: AGGGGACCCACTAATTTTTCTGT<br>R: GTTTCTGCTTCAAGGCTTCCCAT	96-134	HEX	0.2	2	Tsuda *et al*.(2008a)	25	0.712	0.718	0.784	0.076	0.012
Bet50	AF310854	(CT)12CCTT(CT)4	F: GTTTTGGGTTTCCACTTCCA<br>R: ACTGGTAATACCTTTACCAAGCC	134-158	FAM	0.2	2	Kulju *et al*. (2004)	10	0.638	0.618	0.71	0.13	-0.021
Bet31	CD276907	(TC)8	F: AGGTTGCTCAACCTAACCAACAT<br>R: GTTTGGACAAGAACAACCACCAAG	220-232	FAM	0.4	2	Tsuda *et al*.(2008a)	11	0.656	0.751	0.842	0.109	0.126
Bet3	CD277113	(TC)9	F: ACAATATCTCACAAATCTGCCGC<br>R: GTTTAACCTGAACGTCCTCAAAGGTCC	264-286	HEX	0.4	2	Tsuda *et al*.(2008a)	13	0.49	0.47	0.538	0.129	-0.041
Bet38	EE595358	(AG)7T(GA)3CAA(AG)3	F: AGGGGATCCAATTCACAGATACA<br>R: GTTTCGATCGAATTTGAATCCGAAGAAG	128-220	FAM	0.2	3	Tsuda *et al*.(2008b)	17	0.542	0.607	0.668	0.088	0.11
Bet43	AF310851	(CT)3CC(CT)2CC(CT)13AT(CT)5	F: CTCCTTAGCTGGCACGGAC<br>R: CCCTTCTTCATAAAACCCTCAA	208-252	HEX	0.2	3	Kulju *et al*. (2004)	19	0.676	0.657	0.76	0.131	-0.015
Bet48	AF310862	(TC)26	F: AAGGGCACCTGCAGATTAGA<br>R: AAAATTGCAACAAAACGTGC	228-284	TET	0.2	3	Kulju *et al*. (2004)	37	0.717	0.784	0.89	0.117	0.082

Table S2 Prior distributions of the parameters used in DIYABC

Parameter	Minimum	Maximum
Effective population size		
XJ	10	10000
XJa	10	10000
NE	10	10000
NEa	10	10000
NC	10	10000
NCa	10	10000
NW	10	10000
NWa	10	10000
YN	10	10000
YNa	10	10000
NA	10	10000
Time scale in generations [a]		
t1	10	10000
t2	10	10000
t3	10	10000
ta	10	10000
Admixture		
ra	0.001	0.999
Mutation model		
Mean mutation rate	1×10^{-4}	1×10^{-3}
Individual locus mutation rate	1×10^{-5}	1×10^{-2}
Mean coefficient P	1×10^{-1}	3×10^{-1}
Individual locus coefficient P	1×10^{-2}	9×10^{-1}
Mean SNI rate	1×10^{-8}	1×10^{-5}
Individual locus SNI rate	1×10^{-9}	1×10^{-4}

[a] Conditional constraints: ta < t1, ta < t2, t1 < t2, t2 ≤ t3.

Table S3 Parameter estimates of the most likely scenarios obtained by DIYABC. NA, ancestral effective population size; t1, t2, estimated times of the different events depicted in Fig. 3.

Parameter	mean	median	mode	2.5%	5%	25%	75%	95%	97.5%
**Step 1 XJ and NE were both refugia**									
NE	7370.0	7520.0	7950.0	3960.0	4560.0	6370.0	8500.0	9610.0	9780.0
XJ	6740.0	6870.0	7550.0	2830.0	3330.0	5290.0	8350.0	9620.0	9790.0
t1	3390.0	2920.0	2190.0	718.0	903.0	1860.0	4530.0	7530.0	8450.0
NA	3250.0	2600.0	823.0	125.0	234.0	1140.0	4900.0	8420.0	9100.0
**Step 2 Admixture in NC from NE and NW**									
NE	8780.0	9000.0	9530.0	6300.0	6860.0	8300.0	9520.0	9910.0	9950.0
NC	6330.0	6400.0	6350.0	2420.0	2950.0	4840.0	7890.0	9480.0	9720.0
NW	4560.0	4240.0	3290.0	1300.0	1630.0	2920.0	5940.0	8690.0	9240.0
t1	75.1	63.4	55.3	17.0	21.4	41.8	92.4	168.0	209.0
ra	0.383	0.356	0.370	0.034	0.063	0.229	0.508	0.814	0.899
t2	983.0	739.0	515.0	178.0	224.0	455.0	1210.0	2520.0	3200.0
NA	2000.0	1200.0	171.0	54.2	95.8	476.0	2770.0	6840.0	8210.0
**Step 3 NW and YN were both refugia**									
NW	8210.0	8540.0	9890.0	4640.0	5280.0	7400.0	9380.0	9880.0	9940.0
YN	2880.0	2530.0	1860.0	730.0	906.0	1730.0	3610.0	6220.0	7410.0
t1	1180.0	917.0	727.0	211.0	278.0	578.0	1470.0	2970.0	3710.0
NA	945.0	459.0	14.1	23.7	38.6	178.0	1060.0	3650.0	5370.0

Table S4 Correlation analysis of reserved variables

	bio07	bio08	bio15	bio19	bio27	bio32	bio36
bio07	1.000	-0.248	0.096	-0.343	-0.693	-0.482	-0.359
bio08	-0.248	1.000	-0.066	0.134	0.455	0.136	-0.324
bio15	0.096	-0.066	1.000	-0.580	-0.092	-0.307	0.016
bio19	-0.343	0.134	-0.580	1.000	0.256	0.608	0.059
bio27	-0.693	0.455	-0.092	0.256	1.000	0.283	0.046
bio32	-0.482	0.136	-0.307	0.608	0.283	1.000	0.326
bio36	-0.359	-0.324	0.016	0.059	0.046	0.326	1.000

min correlation (bio36 ~ bio15): 0.016
max correlation (bio27 ~ bio07): -0.693

Table S5 VIFs of the remained variables

	Variables	VIF
1	bio07	2.707
2	bio08	1.535
3	bio15	1.546
4	bio19	2.277
5	bio27	2.394
6	bio32	1.993
7	bio36	1.584

Table S6 Ecological niche Model accuracy evaluation

	Testing.data	Cutoff	Sensitivity	Specificity
TSS	**0.798**	325	95.161	84.56
SR	0.306	983	13.71	99.64
ROC	**0.953**	330.5	95.161	84.86
KAPPA	0.267	913	40.323	98.15
ACCURACY	0.988	993	0.806	99.99

Note S1 Speculations about the populations outside China

Two populations of white birch outside China had fewer samples. The Russian Far East (RS) population had a genetic composition similar to that of the NE lineage (Fig. 3), and in the past, the northern population seemed to have a continuous distribution, from which we can infer that the population from Russia belonged to the NE genealogy, rather than representing an independent one. FJ from Japan had similar genetic composition with NE, also containing quite a few components from other gene pools. There are two possible explanations. One is that the FJ population maintained ancestral polymorphism, after the geographical segregation with the continental populations; the other is that there were individual migrations through a land bridge from the continent to the Japanese islands during the LGM period when the sea level was lower than at present. However, considering that FJ is a population containing only 15 individuals, and is located quite near to the Fujiyama scenic spot, the possibility of an introduction from other locations by visitors and pilgrims cannot be ruled out.
